# Assessment of BCG and inactivated *Mycobacterium bovis* vaccines in an experimental tuberculosis infection model in sheep

**DOI:** 10.1371/journal.pone.0180546

**Published:** 2017-07-05

**Authors:** Ana Balseiro, Raúl Altuzarra, Enric Vidal, Xavier Moll, Yvonne Espada, Iker A. Sevilla, Mariano Domingo, Joseba M. Garrido, Ramón A. Juste, Miguel Prieto, Bernat Pérez de Val

**Affiliations:** 1 SERIDA, Servicio Regional de Investigación y Desarrollo Agroalimentario, Centro de Biotecnología Animal, Gijón, Asturias, Spain; 2 Departament de Medicina i Cirugia Animals, Universitat Autònoma de Barcelona, Bellaterra, Barcelona, Catalonia, Spain; 3 IRTA, Centre de Recerca en Sanitat Animal (CReSA, IRTA-UAB), Campus de la Universitat Autònoma de Barcelona, Bellaterra, Barcelona, Catalonia, Spain; 4 NEIKER-Tecnalia, Instituto Vasco de Investigación y Desarrollo Agrario, Departamento de Sanidad Animal, Derio, Bizkaia, País Vasco, Spain; 5 Departament de Sanitat i Anatomia Animals, Universitat Autònoma de Barcelona, Bellaterra, Barcelona, Catalonia, Spain; Fundació Institut d’Investigació en Ciències de la Salut Germans Trias i Pujol, Universitat Autònoma de Barcelona, SPAIN

## Abstract

**Background/Aims:**

Animal tuberculosis (TB) is a complex animal health problem that causes disruption to trade and significant economic losses. TB involves a multi-host system where sheep, traditionally considered a rare host of this infection, have been recently included. The aims of this study were to develop an experimental TB infection model in sheep with a *Mycobacterium caprae* field strain isolated from a tuberculous diseased ewe, and to use this to evaluate the safety and efficacy of two vaccines against TB in sheep, the live-attenuated *M*. *bovis* BCG vaccine (Danish strain) and a heat-inactivated *M*. *bovis* (HIMB) vaccine.

**Methods:**

Eighteen 2 month-old lambs were experimentally challenged with *M*. *caprae* by the endotracheal route (1.5 × 10^3^ CFU). They were separated per treatment group into parenterally vaccinated with a live BCG Danish strain vaccine (*n* = 6), orally vaccinated with a suspension of HIMB (*n* = 6) and unvaccinated controls (*n* = 6). Clinical, immunological, pathological and bacteriological parameters of infection were measured.

**Results:**

All lambs were successfully infected and developed gross TB lesions in the respiratory system. The BCG vaccine conferred considerable protection against experimental TB in lambs, as measured by a reduction of the gross lesion volumes and bacterial load. However, HIMB vaccinated animals did not show protection.

**Conclusions:**

This study proposes a reliable new experimental model for a better understanding of tuberculosis in sheep. BCG vaccination offers an effective prospect for controlling the disease. Moreover alternative doses and/or routes of administration should be considered to evaluate the efficacy of the HIMB vaccine candidate.

## Introduction

Animal tuberculosis (TB) is a mycobacterial disease that affects a broad range of mammals including domestic animals, wildlife and humans. It is caused by members of the *Mycobacterium tuberculosis* complex (MTBC), most commonly *Mycobacterium bovis* and *Mycobacterium caprae* [1]. It is one of the most complex animal health problems that the ungulate farming industry faces today [2]. TB causes disruption to trade and significant economic losses [3]. In Europe animal TB involves a multi-host system where domestic (cattle and goats) and wildlife (badger, wild boar and red deer) have a relevant role that varies between regions, depending on climate, habitat and husbandry factors [4]. Recent studies have shown that sheep, traditionally considered a rare host for the MTBC, can be part of the multi-species system which may maintain TB in a region, at least in mixed farms where sheep cohabit with TB-infected cattle and/or goats [5]. Given the abundance and widespread distribution of domestic sheep in Europe and elsewhere, that role may have important implications for global animal TB control, particularly since sheep are not routinely subjected to the periodic diagnostic testing within the National Programmes to eradicate bovine TB in many countries where TB is prevalent in cattle or goats [5].

Vaccination against TB gains renewed interest when the strategy of test and cull fails in achieving TB eradication. Nowadays, bacille Calmette-Guérin (BCG), a live attenuated strain of *M*. *bovis*, is the only available vaccine against human TB and its efficacy and safety have already been studied in several animal TB hosts, such as cattle, goats, badgers, brushtail possums, wild boar, white-tailed deer and African buffalo [6, 7, 8, 9, 10, 11, 12, 13, 14, 15, 16]. However, the use of BCG vaccination against *M*. *bovis* in cattle is explicitly forbidden by EU legislation. The rationale is the lack of availability of diagnostic tests to differentiate vaccinated from infected animals (DIVA tests) [2]. In countries where test and cull control strategies are ongoing, DIVA tests would be necessary if, vaccination with BCG or other mycobacterial based vaccines were applied [17]. Moreover, regarding wildlife oral vaccination, the safety of baits on non-target species must be also considered [2]. Thus, a promising strategy is the use of new inactivated oral vaccine candidates that display protection against TB and would also have the advantages of being environmentally more stable and safe under field conditions and of minimizing cross-reactions in diagnostic tests [18].

The aims of this study were (i) to develop an experimental TB infection model in sheep with a *M*. *caprae* field strain isolated from a tuberculous diseased ewe, and (ii) to use it to evaluate the efficacy of two vaccines against TB in sheep, a live BCG vaccine (Danish strain) and a heat-inactivated *M*. *bovis* preparation.

## Material and methods

### Animals and experimental design

Eighteen 2 month-old Lacaune male lambs obtained from a farm with no TB history were selected on the basis of negative results to the Interferon Gamma (IFN-γ) assay (ID Screen^®^ Ruminant IFN-g, ID.vet, Grabels, France) and were housed on an experimental farm where they were identified and either parenterally vaccinated with a live-attenuated *M*. *bovis* BCG Danish 1331 strain vaccine (*n* = 6) or orally vaccinated with a suspension of heat-inactivated *M*. *bovis* (HIMB) (*n* = 6). The remaining 6 animals were kept as unvaccinated controls. Six weeks after vaccination lambs were moved and housed in biosafety level 3 containment facilities for acclimatization for one week prior to experimental challenge with *M*. *caprae*, which was performed at week 7.

Before and during the experimental infection the lambs were observed for clinical signs. Rectal temperature and body weight were measured prior to challenge and weekly and every two weeks after challenge, respectively. Blood samples were collected at weeks 0, 2, 4, 7, 9, 11, 13, 15, 17 and 19 of the experiment from the jugular vein in heparinized blood tubes. Sampling procedures and the challenge method were approved by the Animal Research Ethics Committee of the Principado de Asturias, Spain (reference number PROAE 20/2015).

### Preparation of *M*. *bovis* BCG vaccine

The *M*. *bovis* BCG inoculum was prepared as previously described [14]. Briefly, *M*. *bovis* BCG Danish 1331 strain (ATCC, Ref. 35733^™^) was sub-cultured in Middlebrook 7H9 media (BD Diagnostics, USA). The bacterial growth was processed and titered as described above. The stock suspension was thawed and diluted to 10^6^ colony forming units (CFU)/ml in sterile PBS. A single dose of 0.5 ml of this suspension (5 × 10^5^ CFU) was subcutaneously inoculated on the right scapula.

### Preparation of heat-inactivated *M*. *bovis* vaccine (HIMB)

The *M*. *bovis* strain used was first isolated from a naturally infected wild boar on Coletsos medium [18]. The isolate was propagated in Middlebrook 7H9 broth enriched with Oleic acid-Albumin-Dextrose-Catalase (OADC Enrichment; Difco, USA) for 2–3 weeks. Cells were harvested by centrifugation at 2,500 x g for 20 minutes and washed twice in PBS. Bacterial pellets were suspended in PBS and declumped using a fine needle syringe. After adjusting the bacterial concentration, serial dilutions of the suspension were plated to assess the number of CFU per milliliter. Inactivation of bacteria was carried out in an airtight bottle submerged in a water bath at 84–85°C for 45 minutes. Oral doses consisted of 2 ml of the inactivated suspension containing approximately 10^7^ CFU/ml of heat-inactivated bacteria. Inactivation was confirmed by culturing ten doses of the suspension in Mycobacterial Growth Indicator Tubes (MGIT; Becton Dickinson, USA) and ten in agar-solidified Middlebrook 7H9 plates. The oral vaccine was administered using a 2 ml syringe at week 0 of the experiment.

### *M*. *caprae* inoculum preparation

The *M*. *caprae* SB0157 (www.Mbovis.org) field strain used as inoculum was originally isolated from a tuberculous sheep from Catalonia. The isolate was sub-cultured in Middlebrook 7H9 broth (BD Diagnostics, Sparks MD, USA) supplemented with 0.5% (v/v) Tween 80, 40 mM sodium pyruvate (Sigma-Aldrich, Steinheim, Germany) and 10% (v/v) albumin dextrose catalase enrichment (BD Diagnostics, USA) at 37°C for 21 days. The culture was centrifuged at 2500 rpm for 20 minutes and suspended in 2 ml of phosphate-buffered saline (PBS; Sigma-Aldrich, Steinheim, Germany) with 0.05% Tween 80 (PBS-T80). Two 1 ml aliquots of this stock suspension were stored at -70°C. Then an aliquot of the stock suspension was thawed and titered by plating 100μl ten-fold serial dilutions in 7H11 solid media (BD Diagnostics, USA). Plates were incubated at 37°C and CFU were counted after 28 days. The inoculum was prepared using the other aliquot at the required final suspension of 1.5 × 10^3^ CFU of *M*. *caprae* by diluting the stock suspension with PBS.

### Experimental challenge

For infection at week 7, lambs were first sedated with 0.05 mg/kg of acepromazine maleate (Equipromacina^®^) and 0.2 mg/kg of butorphanol tartrate (Torbugesic^®^) co-administered by intramuscular injection. Approximately 30 minutes later, lambs were anesthetized with 4–6 mg/kg of propophol (Propofol Lipuro^®^) and 0.2 mg/kg of midazolam (Dormicum^®^) administered intravenously. Afterwards, lambs were intubated with a sterilized endotracheal tube and were placed in right lateral decubitus. A plastic cannula (3.3 mm outer diameter) was passed through the endotracheal tube to the level of the carina. For inoculation, a thinner cannula (2.1 mm outer diameter) was passed through the thicker one to a bronchus, and then 0.5 ml of *M*. *caprae* inoculum was injected into the inner cannula, followed by flushing with 1 ml of 0.9% saline. The lambs recovered from anesthesia in sternal decubitus.

### Antigens and peptides

Bovine purified protein derivative *M*. *bovis* (PPD-B) and *M*. *avium* (PPD-A) tuberculins were obtained from CZ Veterinaria (Porriño, Galicia, Spain). Recombinant proteins ESAT-6, CFP-10 and MPB83 were obtained from Lionex (Braunschweig, Germany). Peptide cocktails Prionics^™^ PC-HP (based on ESAT-6, CFP-10 and 4 additional mycobacterial antigens) and Prionics^™^ PC-EC (based on ESAT-6 and CFP-10) were supplied by Thermo Fisher Scientific (Schlieren, Switzerland). Pokeweed mitogen (PWM) was obtained from Sigma-Aldrich.

### Skin tests

Single intradermal tuberculin (SIT) and comparative intradermal tuberculin (CIT) tests were carried out on all lambs at 10 weeks post-challenge (p.c.) (week 17 of the experiment) by inoculating 0.1 ml (2500 IU) of PPD-B and PPD-A on the right and the left side of the neck, respectively. Skin-fold thickness was recorded just before inoculation and after 72 h. Qualitative results were read according to the standard interpretation used in bovine TB eradication programs (Council Directive 64/432/EEC) [19]. Lambs were considered positive by SIT test when clinical signs at the PPD-B inoculation site were observed or if the increase in skin-fold thickness (SFT) at the PPD-B inoculation site was ≥4 mm, and inconclusive if SFT was >2 mm and <4 mm. Lambs were considered positive by CIT test when clinical signs at the PPD-B inoculation site were observed or if the increase in SFT at the PPD-B inoculation site was >4 mm and higher than the increase at the PPD-A inoculation site, and inconclusive when the SIT test was positive or inconclusive and SFT at the PPD-B inoculation site was >1 mm and ≤4 mm higher than the increase at the PPD-A inoculation site.

### Whole-blood IFN-γ assay

Blood samples were collected at the time points described above, preserved at room temperature, and processed less than 2 h after collection as described previously [16]. Briefly, one milliliter of whole blood was stimulated in 96-deepwell cell culture plates (Eppendorf Ibérica, Madrid, Spain) with PPD-B, PPD-A (both used at a final concentration of 20 μg/ml), a mixture 1:1 of recombinant proteins ESAT-6 and CFP-10 (E/C), used at a final concentration of 10 μg/ml, and peptide cocktails PC-HP and PC-EC, used at weeks 0, 7, 9, 11, 13, 15 and 17 of the experiment at the provider´s recommended concentration. Blood samples stimulated with PWM (at a final concentration of 10 μg/ml) or PBS were used as positive and negative controls, respectively. Samples were subsequently incubated at 37°C overnight and ruminant IFN-γ ELISA (ID Screen^®^ Ruminant IFN-g) was conducted from plasma supernatant following the manufacturer instructions. The interpretation of qualitative results for the standard IFN-γ assay (PPD-B and PPD-A) were performed according to manufacturer’s instructions.

### Serology

Plasma samples from all animals were analyzed in duplicate for antibodies to MTBC using a home-made ELISA [20]. 96-well plates were coated with the cell-surface lipoprotein MPB83 (0.5 g/ml) diluted in carbonate/bicarbonate buffer and incubated overnight at 4°C. After blocking for 45 minutes at 37°C with PBS containing 0.05% Tween 20 (PBS-T20) with 0.5% casein, plasma samples (at 1/200 dilution in PBS-T20 with 1% casein) were added in duplicate and incubated for 1 hour at 37°C. After washing, a combination of protein A and protein G conjugated with peroxidase (Sigma-Aldrich, Steinheim, Germany) was added at final concentrations of 50 ng/ml and 100 ng/ml, respectively. The plates were then read in a spectrophotometer, and the OD450 was measured. A sample was classified as positive when the ΔOD450 (sample OD450 minus the blank wells OD450) was equal or higher than the optimal cut-off point (ΔOD450 = 0.2) as previously determined [20].

### Post-mortem examination

All lambs were euthanized at 12 weeks p.c. (week 19 of the experiment) by intravenous injection of sodium pentobarbital. At necropsy, lung gross lesions were recorded first by palpation and external visual inspection was performed. Visible TB lesions found in other organs were also recorded. Retropharyngeal (right and left), bronchial (right and left) and mediastinal (cranial, medial and caudal) whole lymph nodes (LNs) were carefully removed without incising the pleura and after this, lesion volumes were measured and processed for bacterial count. The whole lungs (filled with formalin), submandibular and preescapular LNs were fixed with 10% buffered formalin as previously described [21]. One month later the lungs were processed by computed tomography (CT). To determine the volume of TB lesion in the LNs, the number and diameter of macroscopic lesions were recorded and the approximate volumes of gross lesions were calculated as the most likely 3D-geometrical morphology (namely, sphere, cylinder or prism). Submandibular and preescapular LNs were assessed by histopathology—hematoxylin and eosin and Ziehl-Neelsen (ZN) staining specific for acid-fast bacilli. Any other tissue showing any TB compatible lesion was also submitted to histopathological analysis.

### Computed tomography (CT)

After complete fixation, the lungs were scanned using a 16-slice multi-detector CT scanner (Brivo CT-385, GE Healthcare, UK). Scanning parameters were: 120 kV, 122 mAs, with a large sample field of view (SFOV: 43cm) and 0.6 mm slices using soft tissue and lung algorithms. Sequential slices were analyzed and afterwards processed with the CT software allowing calculation of the volume. Tuberculous lesions were defined as any lesion in the lung parenchyma with different density patterns: calcified lesions, cavitary lesions and solid lesions. The total pulmonary volume, total volume of TB lesions and the total volume of the calcified lesions were calculated for each lung. The total pulmonary volume was calculated with volume rendering (VR). To calculate the volume of the TB lesions 2D, 3D images, VR and multiplanar reconstructions were used and with segmentation tools they were isolated from the adjacent parenchyma. Within the TB lesions, the calcified ones were selected by their Hounsfield Units (range 100-400HU) and the total volume of them were calculated.

### Bacterial count

Pulmonary and retropharyngeal LNs were homogenized in 10 ml of sterile distilled water in a homogenizer (Masticator, IUL Instruments, Barcelona, Spain). The homogenate was decontaminated with a final concentration of 0.35% w/v hexadecylpyridinium chloride (HCP) for 15 minutes in orbital shaking and then centrifuged at 2471 × g for 30 minutes. The supernatant was discarded and the pellet was suspended in 10 ml of PBS-T80. Homogenates were ten-fold serially diluted in sterile PBS and 0.1 ml of each dilution was plated on Middlebrook 7H11 medium. The inoculated plates were incubated at 37°C for 28 days. Afterwards, CFU enumeration was performed and the total bacterial load in LNs was inferred. Colonies were confirmed as MTBC by multiplex PCR [22].

### Data analysis

Comparisons of rectal temperature, weight increase and bacterial load (Log_10_-transformed counts) in LNs among the experimental groups were performed by one-way ANOVA with Tukey test for pairwise comparisons. Differences in antigen-specific IFN-γ and IgG responses, as well as differences in volumes of gross lesions among experimental groups, were compared by non-parametric Kruskal-Wallis test with the *post hoc* Wilcoxon rank sum test. Correlations between antigen-specific IFN-γ responses and post mortem parameters were assessed by non-parametric Spearman's rank correlation coefficient. Significance was set at *p*-value < 0.05. Statistical analysis was conducted with R package v2.15.0 (R Foundation for Statistical Computing, Vienna, Austria).

## Results

### Clinical signs and body condition

All animals were observed daily throughout the experiment and TB symptoms were not detected. Rectal temperature was measured weekly p.c.

**Fig 1:**
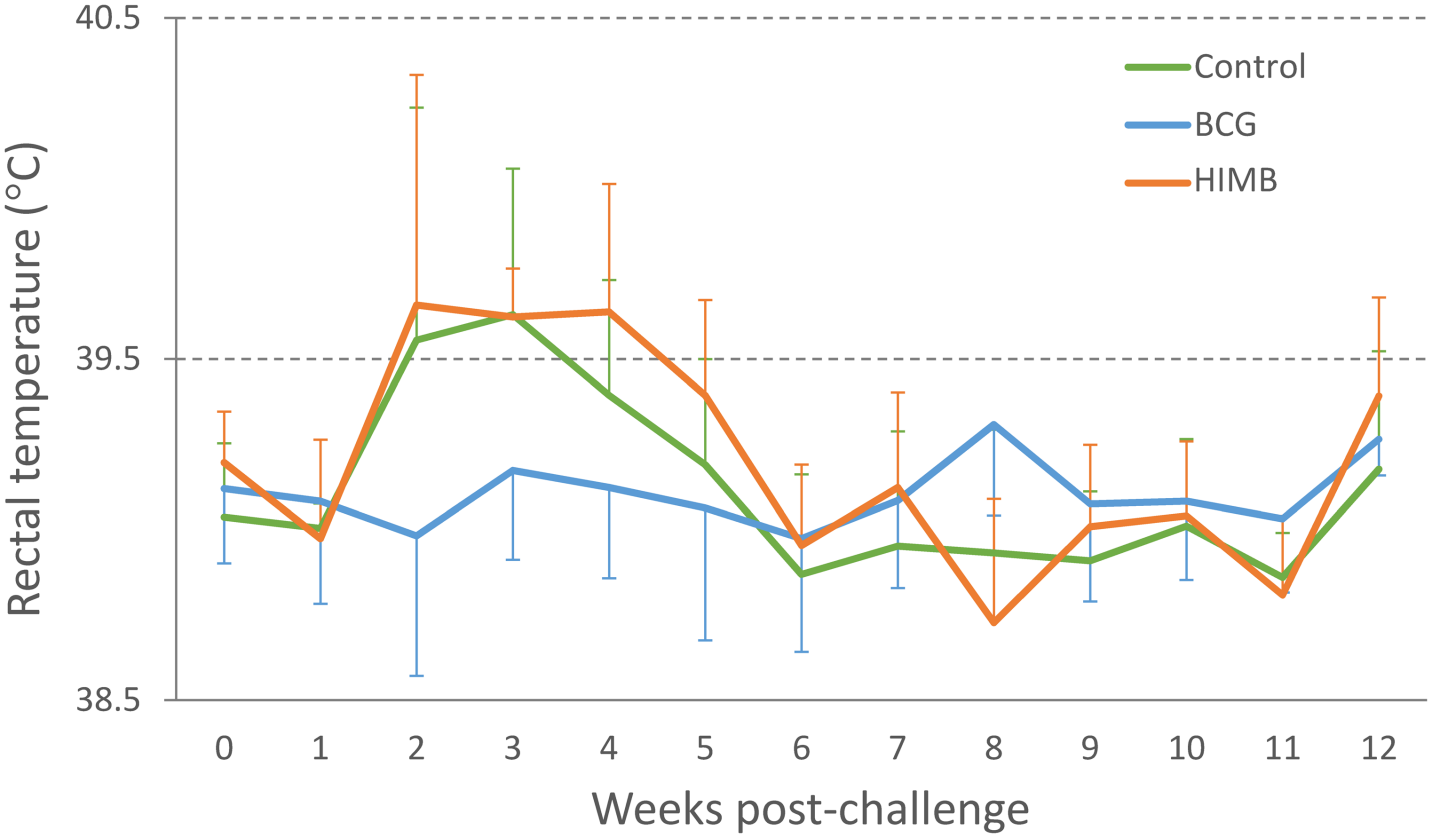
Rectal temperature after *M*. *caprae* challenge. Results are expressed as mean temperature (°C) ± 95% confidence interval (CI) in unvaccinated control (n = 6), BCG vaccinated (n = 6) and HIMB vaccinated (n = 6) lambs.

A peak of mean rectal temperature was observed on weeks 2–4 p.c in the control and HIMB vaccinated groups (Fig 1). Rectal temperatures of lambs in the unvaccinated control group were significantly higher (*p* < 0.05) than in the BCG vaccinated group on week 10 p.c. (Fig 1). Rectal temperatures of lambs in the HIMB vaccinated group were significantly higher (*p* < 0.05) than in the BCG vaccinated group from week 10 to 11 p.c. (Fig 1). From week 12 p.c. rectal temperatures remained below 39.5°C, with no statistical differences between the groups except for week 15 when the BCG vaccinated group presented a temperature significantly higher (*p* < 0.05) than the HIMB vaccinated group.

**Fig 2:**
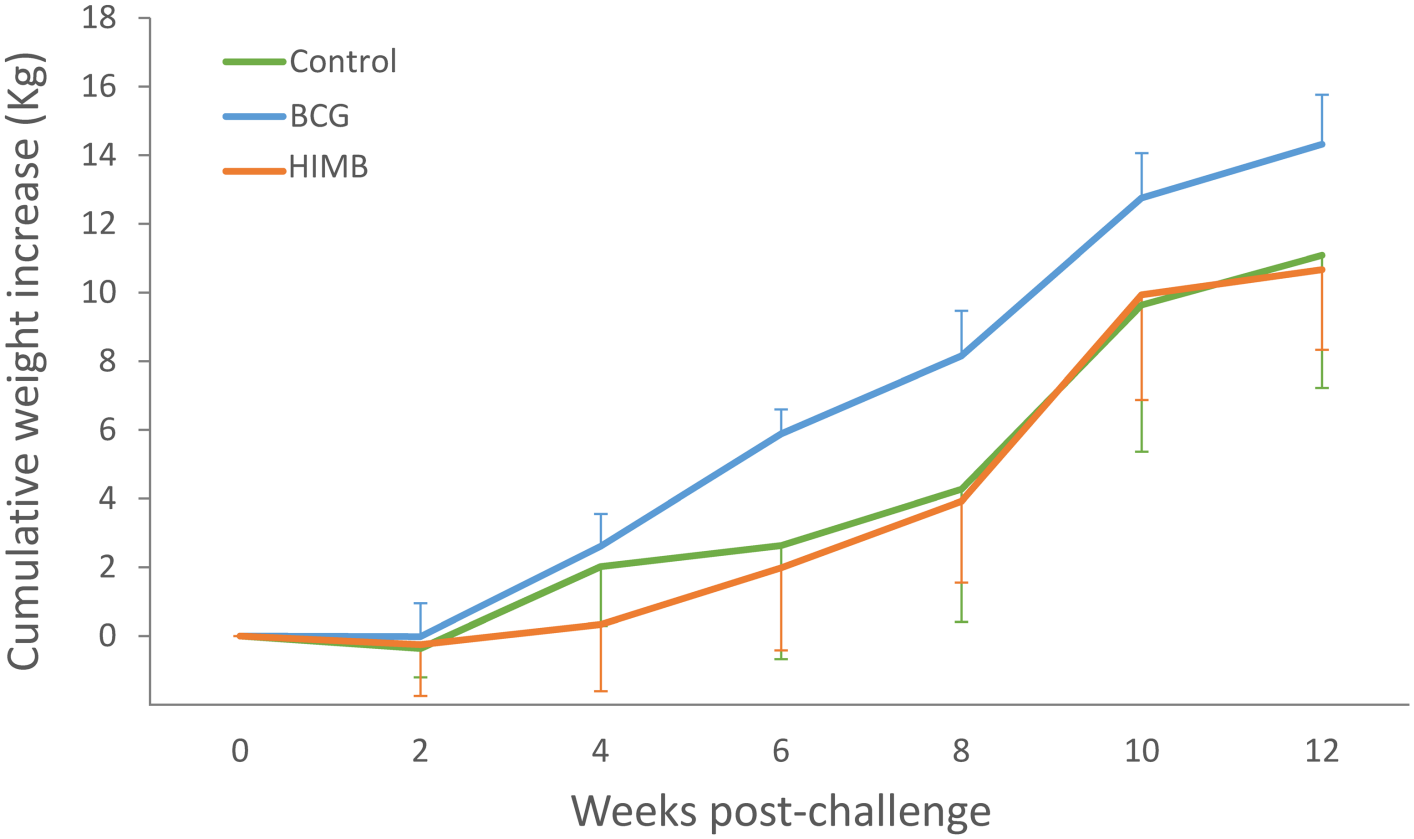
Body weight increase of lambs after *M*. *caprae* challenge. Results are expressed as mean cumulative weight (Kg) ± 95% CI in unvaccinated control (n = 6), BCG vaccinated (n = 6) and HIMB vaccinated (n = 6) lambs.

In the BCG vaccinated group the mean body weight increase was significantly higher (*p* < 0.05) than in the unvaccinated control animals at weeks 6 and 8 p.c. and in the HIMB vaccinated group at weeks 4, 6, 8 and 12 p.c. (Fig 2).

### Immune responses following vaccination and challenge

The immunological response to vaccination and to infection with *M*. *caprae* was characterized using both cell-mediated and humoral immunological tests. Table 1 shows the mean responses per treatment group for SIT and CIT tests. All animals were positive by SIT and all except for two (one BCG and one HIMB) to CIT at week 10 p.c.

**Table 1:** Results of the single intradermal tuberculin (SIT) and comparative intradermal tuberculin (CIT) tests. (+) Positive, (-) Negative, (I) Inconclusive.

Group	Id.	Skin test (week 17, 10 weeks post-challenge)						
		0h		72h		ΔPPD-B- ΔPPD-A	Result	
		PPD-A	PPD-B	PPD-A	PPD-B		SIT	CIT
Control	44	4.66	4.1	10.93	17.51	7.14	+	+
	45	3.61	3.49	6	10.7	4.82	+	+
	46	3.39	3.44	11.26	22.06	10.75	+	+
	48	3.19	3.25	6.95	11.47	4.46	+	+
	49	3.12	3.28	12.63	20.9	8.11	+	+
	66	3.71	3.55	8.87	13.1	4.39	+	+
BCG	50	4.45	4.05	11.14	20.92	10.18	+	+
	51	3.95	3.3	7.52	13.54	6.67	+	+
	52	3.71	3.94	8.41	15.9	7.26	+	+
	56	3.48	3.81	7.75	14.09	6.01	+	+
	57	3.44	3.41	8.48	16.88	8.43	+	+
	61	3.53	3.66	8.43	9.06	0.5	+	-
HIMB	53	3.05	3.11	9.88	14.55	4.61	+	+
	54	3.89	3.63	9.26	14.11	5.11	+	+
	55	3.62	3.6	7.61	12.66	5.07	+	+
	58	4.55	4.29	11.35	13.81	2.72	+	I
	59	3.22	3.02	6.65	19.42	12.97	+	+
	60	4.15	3.76	7.77	19.88	12.5	+	+

**Fig 3:**
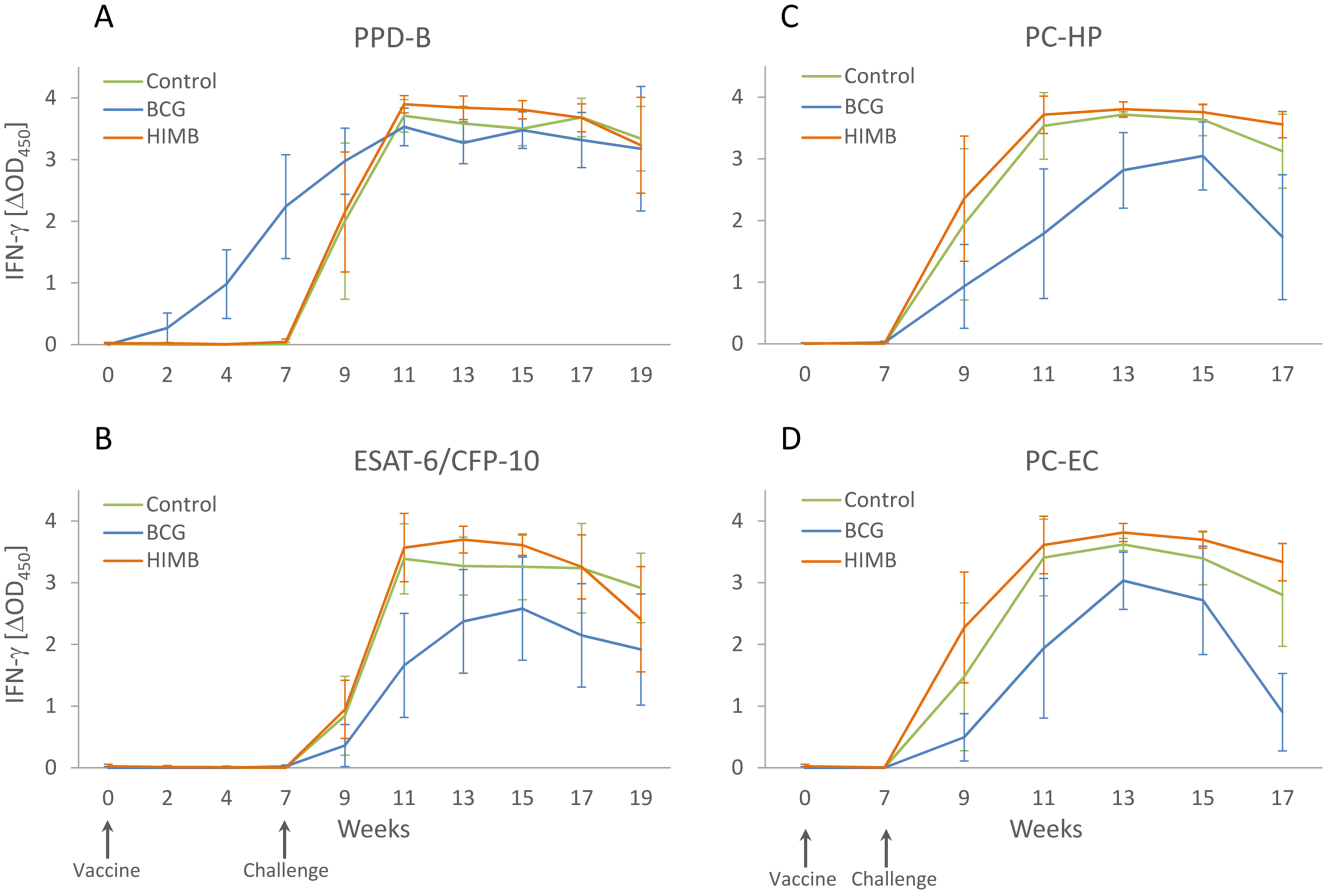
Whole blood antigen specific IFN-γ responses after vaccination and *M*. *caprae* challenge. The graphic shows the determination of the IFN-γ release by ELISA (expressed as ΔOD_450_ ± CI) in plasma supernatants after blood stimulation with (A) *M*. *bovis* PPD (PPD-B), (B) ESAT-6/CFP-10 recombinant antigens mixture, (C) PC-HP cocktail and (D) PC-EC cocktail in unvaccinated control (n = 6), BCG vaccinated (n = 6) and HIMB vaccinated (n = 6) lambs.

Mean IFN- γ *ex vivo* response per treatment group for PPD-B and ESAT-6/CFP-10, PC-HP and PC-EC cocktails is shown in Fig 3. The IFN- γ response to PPD-B started to rise in week 2 p.c. in the BCG group and was significantly higher in this group than in control and HIMB groups (*p* < 0.01) at 2, 4 and 7 weeks of the experiment and significantly lower than the HIMB group (*p* < 0.05) at 13 week of the experiment.

**Fig 4:**
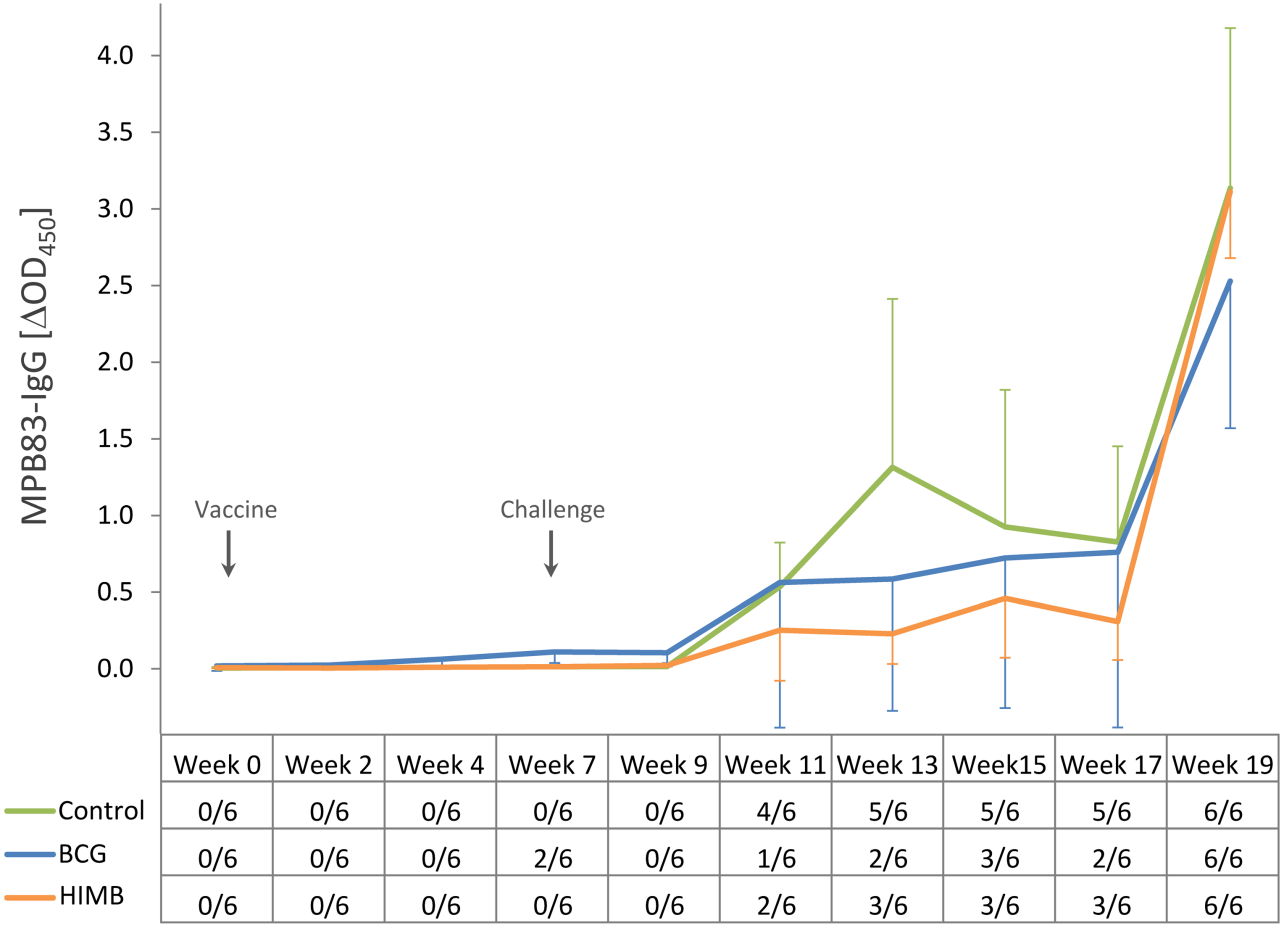
Antibody responses to MPB83 after vaccination and *M*. *caprae* challenge. The graphic shows the MPB83-specific IgG levels determined by ELISA (expressed as ΔOD_450_ ± CI) in unvaccinated control (n = 6), BCG vaccinated (n = 6) and HIMB vaccinated (n = 6) lambs. The table on the horizontal axis shows the qualitative results of the test (No. of seropositive/total lambs) for each group and time point.

Levels of MPB83-specific IgG were measured as the antibody responses to *M*. *caprae* (Fig 4). The BCG group presented slightly higher IgG levels than the other two groups prior to challenge, which were significantly higher at week 7 of the experiment (*p* < 0.05). At 4 weeks p.c. (week 11 of the study), 4 out of 6 control animals and 2 out of 6 HIMB vaccinated animals had already seroconverted. At week 13 of the experiment (6 p.c.) a peak of IgG levels was observed in the control group, significantly higher than in the HIMB vaccinated group (*p* < 0.05). At this time point, 2 BCG vaccinated lambs were positive by the test. Two weeks after the CIT test, levels of IgG rose in three groups and all animals were positive.

### Pathology and bacteriology

**Fig 5:**
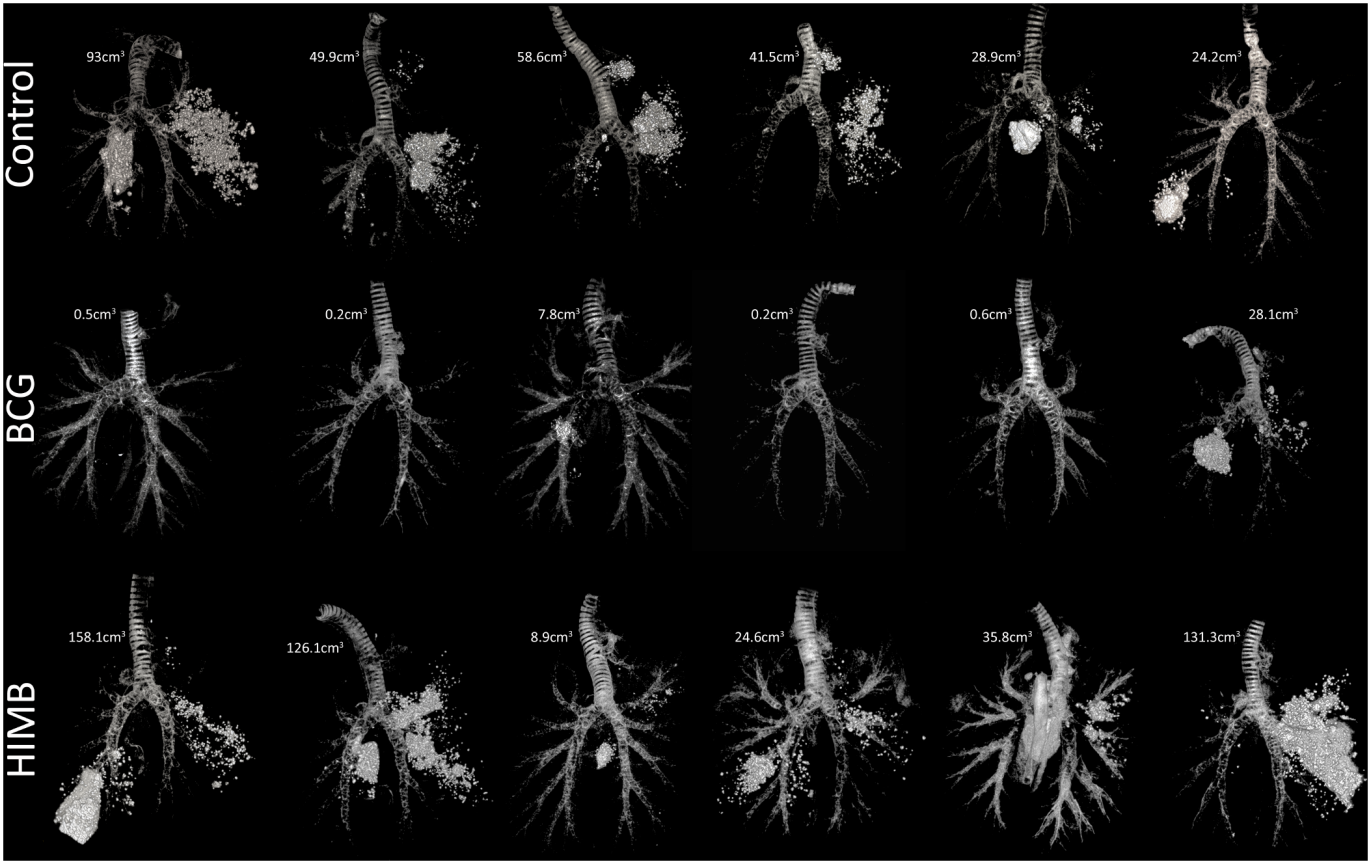
Individual volume of pulmonary gross lesions measured by computed tomography (CT). The figure shows rendering images of the total lesion volume (expressed in cm^3^) for each lung of the 6 unvaccinated control, 6 BCG vaccinated and 6 HIMB vaccinated lambs. The trachea and the bronchial tree are also displayed for anatomical reference.

All lambs presented gross TB lesions at necropsy exhibiting a wide range of sizes when compared between groups. The volume of gross lesions in lungs, the ratio of the volume of lesions/volume of lungs and the volume of calcification in lungs were measured using CT and are shown in Table 2. The BCG vaccinated group showed a significant reduction (*p* < 0.01) of gross lesions (mean volume of gross lesions and ratio of the volume of lesions/volume of lungs, Table 2) in comparison with both the HIMB vaccinated and unvaccinated control groups. A significant reduction in the volume of calcification was also observed in the BCG vaccinated group (*p* < 0.05) compared to the other two groups. The total volume of TB lesions using CT for each animal is shown in Fig 5.

**Table 2:** Pulmonary lesion assessment results for the three experimental groups analyzed by computed tomography (CT). The volume of lung gross lesions, the ratio of the volume of lesions/volume of lungs, and the volume of calcification measured using computer tomography. Number of animals showing cavitary lesions is also shown.

Group	Vol. of lesions (cm^3^)		Ratio Vol. of lesions / Vol. of lungs (%)		Vol. of calcification (cm^3^)		No. of animals with cavitary lesions
	Mean (95% CI)	Median	Mean (95% CI)	Median	Mean (95% CI)	Median	
Control	49.36 (29.41–69.31)	45.73	2.48 (1.21–3.75)	2.33	10.82 (6.24–15.40)	10.03	0/6
BCG	6.20 (0–15.09)	0.53**	0.29 (0–0.72)	0.03**	2.71 (0–7.94)	0.01*	0/6
HIMB	80.80 (29.02–132.58)	80.97	4.83 (1.59–8.07)	4.31	10.41 (5.57–15.25)	9.34	3/6

*p < 0.05;

**p < 0.01;

Kruskal-Wallis test and *post hoc* Wilcoxon rank sum test.

**Fig 6:**
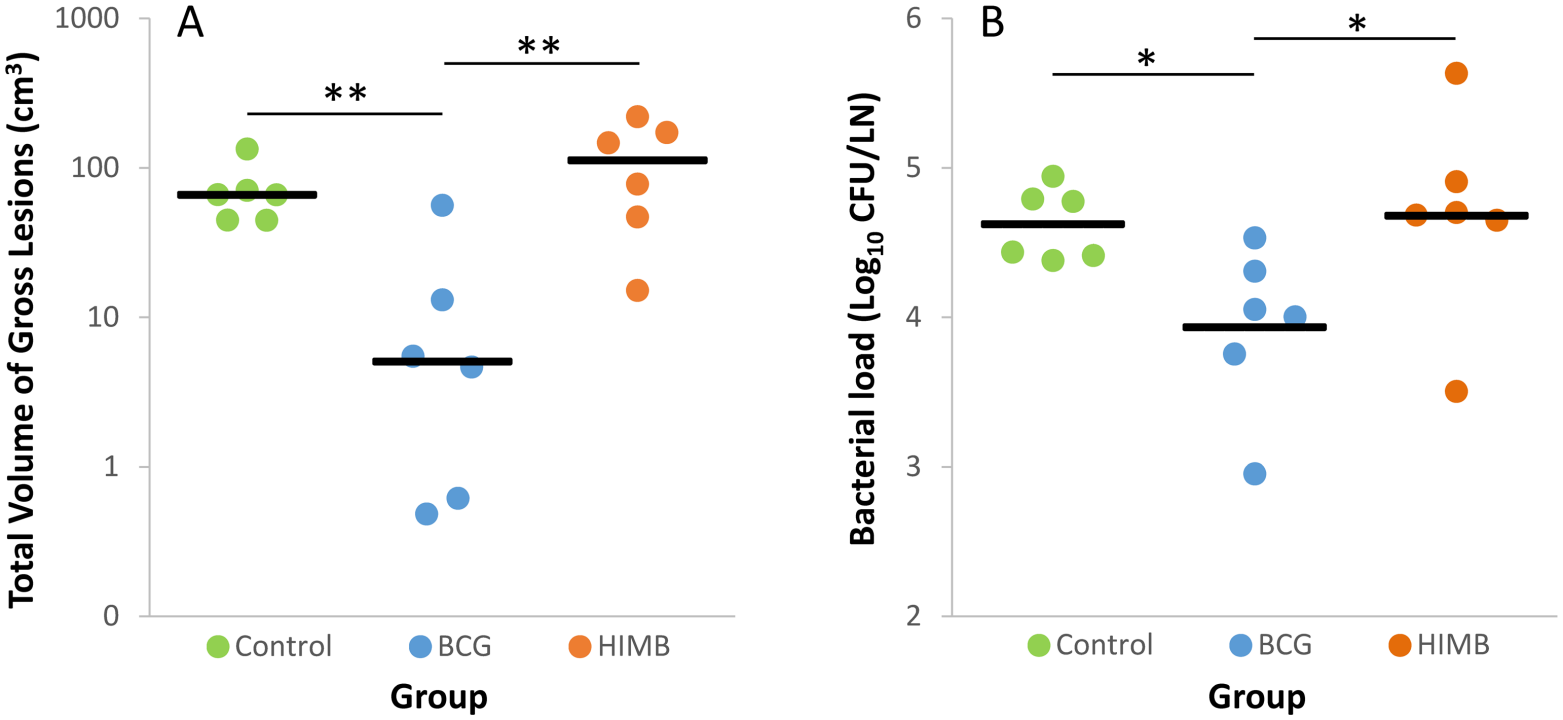
Quantification of gross pathology and bacterial burden of individual lambs. Results are divided according to treatment groups (Control, n = 6; BCG, n = 6; and HIMB, n = 6). (A) Sum of gross lesions volumes (cm^3^) in lungs (measured by CT) and thoracic and retropharyngeal lymph nodes (LN, measured by direct visual examination using a ruler). Horizontal lines indicate median values. **p < 0.01, Kruskal-Wallis test and *post hoc* Wilcoxon rank sum test. (B) Total bacterial load (expressed as Log_10_ CFU) in whole thoracic and retropharyngeal LNs. Horizontal lines indicate mean values. *p < 0.05, one way ANOVA and Tukey *post hoc* test.

The BCG vaccinated group showed a significantly lower mean volume of gross TB lesions in pulmonary and retropharyngeal LNs (7.23 cm^3^, 95% CI: 0–15.64) compared to the HIMB vaccinated group (32.64 cm^3^, 95% CI: 16.77–48.50; *p* < 0.05), and lower but not statistically significant compared to the control group (21.79 cm^3^, 95% CI: 8.57–35.02; *p* > 0.05). BCG vaccinated lambs showed a reduction of the total volume of visible lesions in lungs and respiratory LNs (*p* < 0.01) as well as a reduction of the total bacterial load in respiratory LNs (*p* < 0.05) compared to the other groups (Fig 6).

TB lesions were mainly restricted to the respiratory system although extrapulmonary lesions (generalization) in other LNs were also observed in some animals (see Table 3) and were determined using histopathology. All extrapulmonary TB lesions were positive for the presence of acid-fast bacilli by ZN stain.

**Table 3:** Extrapulmonary lesions (generalization) in non-respiratory lymph nodes (LNs).

Group	Animals showing TB lesions in extrapulmonary LNs				
	Submandibular	Retropharingeal	Preescapular	Hepatic	Jejunal
Control	2/6	1/6	0/6	0/6	1/6
BCG	0/6	0/6	3/6	0/6	0/6
HIMB	2/6	2/6	1/6	1/6	2/6

### Cross-sectional analysis

The association of IFN-γ responses with pathological and bacteriological parameters was evaluated at week 17 (10 weeks p.c. and prior to skin tests). Individual levels of IFN-γ to all antigens showed statistically significant positive correlations with total volume of gross lesions and bacterial load in LNs (Table 4).

**Table 4:** Correlation of Antigen-specific IFN-γ responses at week 17 and post mortem parameters.

	Antigen (IFN-γ levels as ΔOD)			
	PPD-B	ESAT-6/CFP-10	PC-HP	PC-EC
Total gross lesions (cm^3^)	0.5243*	0.5958**	0.6495**	0.8184***
Bacterial load (log CFU)	0.7497***	0.7713***	0.7052***	0.6677**

Tabulated results are Spearman’s rho values.

*p < 0.05,

**p < 0.01,

***p < 0.001.

## Discussion

This is the first report of an experimental model of TB in sheep. All lambs experimentally challenged by the endotracheal route with a low infective dose of *M*. *caprae* were successfully infected and developed gross TB lesions in the respiratory system (100% infection rate). BCG vaccine conferred considerable protection against experimental TB in lambs, as measured by a reduction in the volume of gross lesions and bacterial load in BCG vaccinated animals. However, a single dose of HIMB vaccine did not confer any protection by the oral route at 10^7^ CFU/ml, as neither a reduction in the volume of gross lesions nor bacterial load in respiratory LNs were observed.

It is remarkable that all lambs subjected to experimental challenge became infected, and implies that this study could pave the way to a reliable new experimental model for a better understanding of TB in sheep. Sheep have traditionally been considered as less susceptible to TB infection than other ruminants such as cattle and goats [23, 24]. Nonetheless, this study has shown that the volume of gross pulmonary lesions, quantified by CT, and bacterial load in respiratory LNs in lambs were similar to those observed in goats experimentally challenged with *M*. *caprae* at a similar dose (approximately 10^3^ CFU/ml) [21], suggesting that the susceptibility of sheep to TB infection is similar to that observed in goats.

Macroscopically, lesions in all infected lambs consisted of the typical granulomatous caseous necrotizing lesions in the lung and associated LNs that are usually found in natural cases of TB [5]. The presence of large necrotic coalescing lesions found in unvaccinated control lambs indicated a fast progression of the infection to active TB, similar to what occurs in goats [21]. In contrast, experimental infection with TB in other species such as calves and badgers using the endobronchial route of infection did not cause large coalescent lesions [25, 26], which might indicate a faster progression of TB in lambs compared to these species under similar experimental conditions. Control and HIMB vaccinated lambs showed extrapulmonary lesions in submandibular, retropharyngeal, hepatic, preescapular and mesenteric LNs indicating systemic circulation of mycobacteria, which may lead to excretion and subsequent transmission by inhalatory, oral and fecal routes. Three animals of the BCG vaccinated group showed extrapulmonary TB lesions in the right preescapular LN. This is highly suggestive of drainage of BCG mycobacteria from the point of vaccine inoculation (right scapula) as has been described in the goat model [14].

The efficacy of the parenterally administration of the BCG vaccine in the reduction of clinical signs (pyrexia and prevention of weight loss), gross lesions and the incidence of positive immunological tests in lambs in this study has been also demonstrated in previous studies in different wildlife (wild brushtail possums and badgers) and domestic (cattle and goat) species [6, 12, 14, 27]. The efficacies of different inactivated formulations have been tested with promising results in some cases. In cattle inactivated BCG in a mineral oil adjuvant did not evoke protective immunity [28]. However, the subcutaneous administration of formalin-inactivated BCG mixed with non-phospholipid liposome adjuvants in guinea pigs conferred a significant survival advantage against lethal aerogenous challenge with *M*. *bovis* [29]. Recent studies have also shown that orally administered heat-inactivated *M*. *bovis* vaccine (the same HIMB vaccine used in this study) at a similar dose (6 × 10^6^ CFU/ml) conferred protection against TB in wild boar, responding similarly to BCG [18]. The first explanation for the failure of the HIMB vaccine to protect lambs against TB in this study could be the route of administration. The vaccine might have been damaged in the lambs’ digestive system before being able to induce an effective immune response. The second reason could be that lambs might require a higher dose than the one used in this experimental infection (10^7^ CFU/ml) or even revaccination. Finally the third reason could be the method of challenge that by passes all the natural mucosal and lymphoid barriers overwhelming the local immune response at the bronchial level. However, since one HIMB vaccinated lamb showed values in the volume of gross lesions, bacterial load and cumulative weigh increase similar to the mean values of the BCG group, we might speculate that the HIMB vaccine conferred protection to this animal. This may also be supported by the slightly higher PPD-B specific IFN-γ levels of this animal detected at 7 weeks after vaccination. (ΔOD_450_ = 0.144) compared to the undetectable levels of the rest of the HIMB vaccinated lambs (ΔOD_450_ < 0.1). Future experiments should be carried out to find the optimal route of administration and dose of HIMB inactivated vaccine to induce protection.

The kinetics of whole blood antigen-specific IFN-γ responses is in agreement with the pathological and bacteriological results. The *M*. *caprae* infection induced higher IFN-γ levels either to ESAT-6/CFP-10 or PC-EC and PC-HP peptide cocktails in unvaccinated and HIMB vaccinated animals compared to the BCG group, this being consistent with the vaccination outcome assessed as volume of gross lesions and bacterial load. The association of protection with reduced levels of systemic IFN-γ responses to growth-related mycobacterial antigens was also observed in a number of TB vaccine trials conducted in cattle and goats [17]. All animals responded to the DIVA-based antigen formulations (ESAT-6/CFP-10, PC-EC and PC-HP) after challenge, whereas none of the BCG vaccinated animals responded to these antigens after vaccination, confirming its usefulness for DIVA-based diagnosis [16, 17]. In addition, none of the HIMB vaccinated lambs responded to DIVA antigens prior to challenge. Notwithstanding these animals also failed to respond to PPD-B, therefore, further studies are required to assess the suitability of these antigens as DIVA reagents for HIMB vaccination in sheep. In cattle and deer, oral administration of HIMB also failed to generate a response against either PPD-B or ESAT-6/CFP-10 or PC-EC and PC-HP peptide cocktails [30,31]. Post mortem parameters were compared with IFN-γ responses at week 17 (10 weeks p.c.), once well-established the infection, and prior to skins tests to avoid their potential interference on the IFN-γ assay. The results elucidated that IFN-γ responses to all antigens (PPD-B, ESAT-6/CFP-10, PC-HP and PC-EC) are immunological correlates of disease severity (i.e. gross pathology and bacterial load). *Ex vivo* ESAT-6, ESAT-6-CFP-10 or PPD-B specific IFN-γ responses were previously identified as correlates of disease severity in experimentally infected cattle and goats [17, 21, 32].

Humoral responses were measured followed by IgG ELISA to MPB83, a serodominant cell surface protein of the MTBC that may be recognized at the early stages of the infection [33]. Interestingly, unvaccinated control lambs seroconverted earlier than lambs experimentally infected in a previous study [21], seroconversion rates were 66% vs 9% at 4 weeks p.c. and 83% vs 45% at 6 weeks p.c., respectively, suggesting that MTBC infection in sheep may induce a faster humoral response to the infection. All experimental animals were seropositive at 2 weeks after SIT as a result of the PPD-boosting effect, similarly to those previously found in cattle and goats [21, 32].

In conclusion lambs have been shown to be susceptible to *M*. *caprae* experimental infection showing similar clinical, immunological, pathological and bacteriological parameters of infection to goats. Moreover subcutaneous BCG vaccination would offer an effective prospect for controlling the disease in individual animals and could also reduce the prevalence of TB in infected ovine herds. However, it is important to note that all animals became infected. This means that, at least in this model, longer studies are needed to be sure that the short term effects persist in the long term. Alternative doses and/or routes of administration should be applied to evaluate the positive and/or negative effects of the HIMB vaccine candidate in this experimental model.
